# Globus pallidus/putamen T_1_WI signal intensity ratio in grading and predicting prognosis of neonatal acute bilirubin encephalopathy

**DOI:** 10.3389/fped.2023.1192126

**Published:** 2023-09-29

**Authors:** Minggang Yi, Jing Lou, Ruodi Cui, Jianshe Zhao

**Affiliations:** ^1^ Department of Radiology, Children's Hospital Affiliated to Shandong University, Jinan, Shandong, China; ^2^ Department of Radiology, Jinan Children's Hospital, Jinan, Shandong, China; ^3^ Department of Radiology, Shandong Jinan Municipal Hospital of Traditional Chinese Medicine, Jinan, Shandong, China

**Keywords:** acute bilirubin encephalopathy, bilirubin encephalopathy, globus pallidus, magnetic resonance imaging, neonate

## Abstract

**Purpose:**

This study sought to investigate the relationship between the globus pallidus/putamen T1 weighted image (T_1_WI) signal intensity ratio (G/P ratio) and the acute bilirubin encephalopathy (ABE) in neonates, and to develop a new strategy for the grading and prognosis of ABE based on the G/P ratio.

**Methods:**

A total of 77 full-term neonates with ABE were scored according to bilirubin-induced neurological dysfunction and divided into mild, moderate, and severe groups. Cranial magnetic resonance imaging examinations were performed and the G/P ratio was recorded. The follow-up reexaminations were carried out at 6 months, 1 year, and 2 years after the initial examination. The neonates were then divided into two groups, the good prognosis group and the kernicterus spectrum disorder (KSD) group, according to the evaluation of Gesell Developmental Schedules and Brainstem Audio Electric Potential at 6 months.

**Main findings:**

The differences of G/P ratios were statistically significant, not only among the mild, moderate, and severe ABE groups for the initial examinations but also between the KSD and the good prognosis groups for the follow-up reexaminations. Therefore, the ABE grading model and prognosis predicting model could be established based on the G/P ratio. In the KSD group, the area under the receiver operating characteristic curve of the G/P ratio-based predicting model was 93.5%, the optimal critical point was 1.29, the sensitivity was 88.2%, and the specificity was 93.3%.

**Conclusions:**

The G/P ratio can be used as an indicating parameter for both the clinical grading of neonatal ABE and the assessment of neonatal ABE prognosis. Specifically, the G/P ratio greater than 1.29 indicates a KSD of neonatal ABE.

## Introduction

Bilirubin encephalopathy is a toxicity-induced nerve cell degeneration caused by free bilirubin, with neurological abnormalities as the clinical and subclinical manifestations (1, 2). It is also known as bilirubin toxic encephalopathy and is one of the important factors affecting neonates’ survival rates and quality of life (3, 4). In clinical practice, a diagnosis of neonatal acute bilirubin encephalopathy (ABE) is relatively difficult, because its clinical symptoms may be minimal or even absent, and not all cases of severe hyperbilirubinemia will lead to bilirubin encephalopathy (5–8). Auxiliary diagnoses, such as the determination of brainstem auditory evoked potential, total bilirubin/albumin (B/A) ratio, amplitude-integrated electroencephalography, and cerebrospinal fluid bilirubin concentration, have a certain clinical value (9–12) but lack sensitivity and specificity. Magnetic resonance imaging (MRI) technologies are relatively simple and non-invasive and are the main imaging approaches currently used in the diagnosis of neonatal bilirubin encephalopathy. The characteristic MRI manifestation of ABE is a symmetrical high signal intensity of the bilateral globus pallidus on T_1_WI in the neonatal period, which is an important sign of brain injury caused by hyperbilirubinemia (13, 14). High T2 weighted image (T_2_WI) signals in the bilateral globus pallidus are indicative of very severe bilirubin brain damage, but this is rare in the neonatal period and occurs in older infants (15). It has been reported that ^1^H-MRS can be applied in the diagnosis of early-stage bilirubin encephalopathy (16, 17).

MRI is routinely used in clinical practice to observe abnormally high signal intensities of bilateral globus pallidus on T_1_WI, but this mainly depends on the assessments of the neuroradiologist, based on their experience, without any quantitative indicators, and subsequently remains prone to subjective judgment errors. Moreover, the degree of such abnormally high signal intensity varies among different pediatric patients with ABE. Therefore, we assessed the relationship between the degree of abnormally high signal intensity and the degree of neurological damage in bilirubin encephalopathy by quantifying the changes in signal intensity, which consists of a simple operation with strong reproducibility. We analyzed the MRI data from 77 patients with ABE, calculated the T_1_WI signal intensity of bilateral globus pallidus and putamen, as well as their ratio (G/P ratio), in order to investigate the relationship between the G/P ratio with ABE grouping and prognosis to provide an imaging basis for patients’ clinical diagnoses and prognosis evaluations, and to observe the relationship between the appearance of a high signal on T_2_WI of bilateral globus pallidus and prognosis.

## Materials and methods

### Clinical data

The study subjects were 77 full-term ABE patients treated in the Neonatology Department of our hospital from January 2015 to January 2018. The diagnostic criteria for ABE (18) were pathological jaundice, abnormal brainstem auditory evoked potential, and at least two concurrent neurological symptoms, including poor responsiveness, drowsiness, rejection of milk feeding, screaming, increased limb tone, opisthotonus, binocular gaze, and convulsion. Hypoxic ischemic encephalopathy, intracranial infection, and congenital neurological diseases were excluded. All infants had gestational age ≥38 weeks and a disease course <2 weeks, including 44 males and 33 females. Their age in days was 10.23 ± 4.48 days and their body weight was 3,172 ± 633 g. Among the 77 neonates, 50 were born by a cesarean section and 27 by natural delivery. There was no history of asphyxia or hypoxia at birth. The total serum bilirubin (TSB) levels of all infants were ≥342 μmol/L (18) (a term infant with TSB >220.6 μmol/l is the diagnostic criterion for neonatal hyperbilirubinemia). The bilirubin-induced neurological dysfunction score developed by Bhutani and Johnson-Hamerman (19) was used to evaluate the pediatric ABE patients. A total of 50 pediatric patients had a score of 1–3 points, indicating mild disease, poor feeding, and reduced movements. Eighteen pediatric patients had a score of 4–6 points, indicating moderate disease, mainly manifesting as drowsiness, poor response, changes in muscle tone, and torsion dystonia of the trunk. Nine pediatric patients had a score of 7–9 points, indicating severe disease, manifesting as excessive crying, weak or absent crying, coma, convulsion, apnea, opisthotonus, etc. A follow-up reexamination was carried out 6 months later. A total of 47 pediatric patients were followed up, 20 of whom underwent cranial MRI reexamination. All 47 pediatric patients were assessed for development according to the Gesell Developmental Schedules (20, 21) and Brainstem Audio Electric Potential (BAEP) at 6 months, based on which they were divided into the good prognosis group (30 cases) and the kernicterus spectrum disorder (KSD) group (17 cases). Among the 20 pediatric patients who underwent cranial MRI reexaminations, 12 patients were in the KSD group and eight were in the good prognosis group. Follow-up was continued for the 17 patients in the KSD group, and they underwent cranial MRI reexaminations at the ages of 1 and 2 years. A total of 14 pediatric patients were followed up at the age of 1 year, including 11 pediatric patients who underwent cranial MRI reexaminations, and nine pediatric patients who were followed up at the age of 2 years, all of whom underwent cranial MRI reexaminations.

All procedures performed involving human participants were carried out in accordance with the ethical standards of the Ethics Committee of Jinan Children's Hospital (ETYY-2020218) and with the 1964 Helsinki declaration and its later amendments or comparable ethical standards. Informed consent was waived due to the retrospective nature of the study. The children's personal information has been hidden.

### Examination methods

Using a Philips Achieva 1.5 T superconducting MR scanner (Netherlands) with NV16 head/neck coils, the pediatric patients wore a headset and were laid in a supine position on the examination bed, with the positioning line between their eyebrows. Before the examination, the pediatric patients were sedated with 10% chloral hydrate 0.5 ml/kg orally or 5% chloral hydrate 1.0 ml/kg by rectal enema delivery. Conventional axial and sagittal scans were performed, with the scanning sequences including T_1_WI, T_2_WI, and diffusion weighted imaging (DWI). Scanning parameters were as follows: T_1_WI: TE 15 ms, TR 550 ms, slice thickness 4.0 mm, slice spacing 0.4 mm, and slice number 20; T_2_WI: TE 150 ms, TR minimum, slice thickness 4.0 mm, slice spacing 0.4 mm, and slice number 20; DWI: TE 50 ms, TR minimum, b value 1,000 s/mm^2^, slice thickness 4.0 mm, slice spacing 0.4 mm, and slice number 20. Cranial MRIs were reexamined with the same machine and the same parameters for the enrolled pediatric patients at the ages of 6 months, 1 year, and 2 years.

### Image analysis

**Figure 1:**
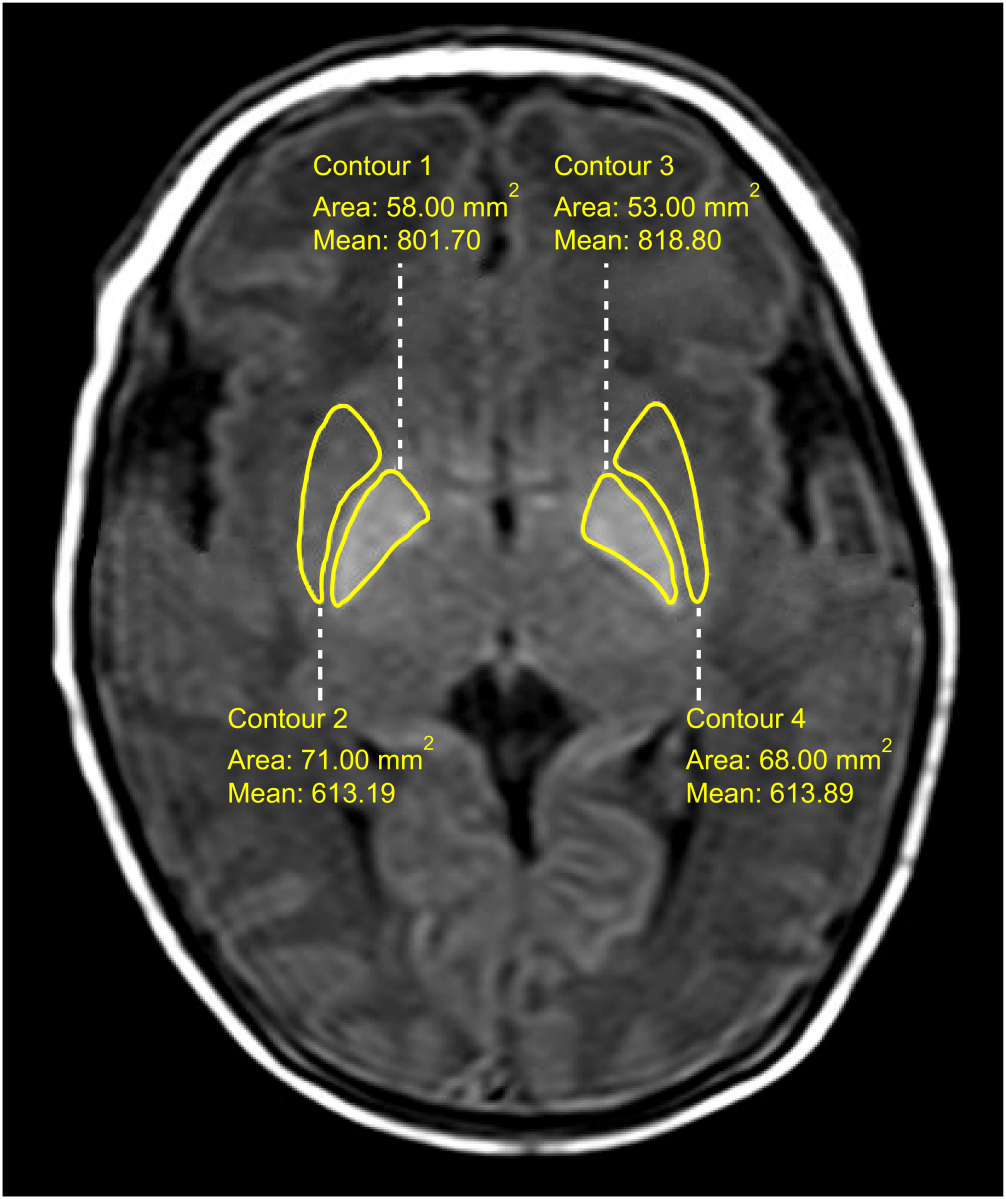
T_1_WI signal intensity measurement of globus pallidus and putamen.

Two radiologists with several years of work experience independently observed the cranial MRI images on a Philips MR mainframe and recorded the ages in days of the pediatric patients at the time of examination. By comparing the globus pallidus signals with the crus posterius capsulae internae and putamen signals in the image, when the globus pallidus T_1_WI and T_2_WI signals were higher than the posterior limb of the internal capsule and putamen signals, respectively, they were considered high signals. A Philips Nebula Workstation was used to measure the T_1_WI signal intensity values of bilateral globus pallidus and putamen on the T_1_WI cross-sectional images. The regions of interest (as shown in Figure 1) were the inner edges of globus pallidus and putamen contours. The G/P ratios were calculated and averaged.

### Statistical methods

GraphPad Prism 6.0 statistical analysis software package was used for the statistical analysis. The bilateral G/P ratios and ages in days of the pediatric ABE patients in the mild, moderate, and severe groups were expressed as $\bar{x}\pm s$. The comparison of G/P ratio and age in days among the mild, moderate, and severe groups was conducted by one-way analysis of variance, and the pairwise comparison among the three groups was conducted by least significant difference (LSD) *post-hoc* analysis. After the test for homogeneity of variance between the good prognosis and KSD groups, an independent samples *t*-test was adopted for comparison and analysis. Prognosis was predicted based on the G/P ratio, and receiver operating characteristic (ROC) curves were plotted to calculate the optimal critical value, sensitivity, specificity, and accuracy. *P* < 0.05 was considered indicative of a statistically significant difference.

## Results

### Observation results of cranial MR images of the pediatric patients

Observation results of cranial MR images of the patients in the neonatal period and the signal intensities on T_1_WI, T_2_WI, and DWI are shown in Table 1.

**Table 1:** Globus pallidus MRI signal manifestations in each ABE group (number of cases).

Group	*N*	T_1_WI	T_1_WI	T_2_WI	T_2_WI
		Hyperintensity^a^	Isointensity	Hyperintensity^b^	Isointensity
Mild ABE	50	29	21	1	49
Moderate ABE	18	12	6	1	18
Severe ABE	9	8	1	2	7

^a^ When the globus pallidus T_1_WI signals were higher than the posterior limb of the internal capsule signals, they were considered high signals.

^b^ When the globus pallidus T_2_WI signals were higher than the putamen signals, they were considered high signals.

**Figure 2:**
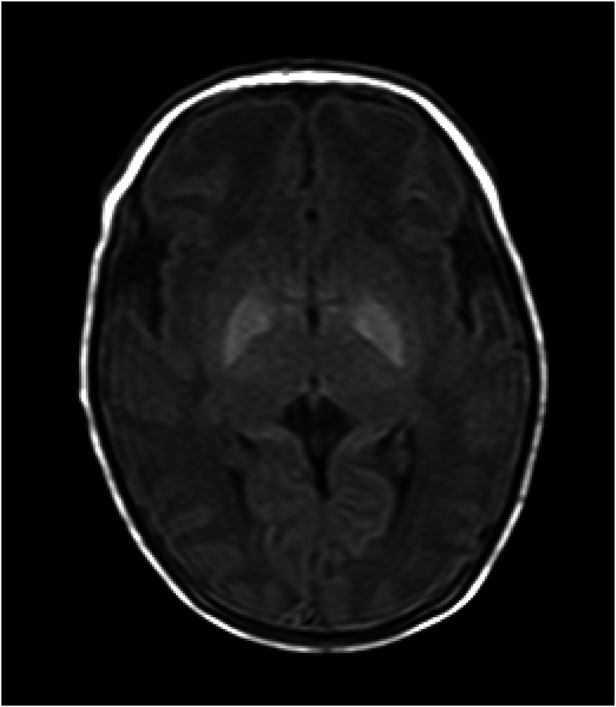
T_1_WI image of the patient. The patient was a 9-day-old female with poor response, small crying sounds, and no convulsions as the main clinical manifestations. The TSB was 402 μmol/L, the bilateral globus pallidus showed significantly high signals on T_1_WI. The T_1_WI signal intensity values of the globus pallidus and putamen were 186 and 141, respectively, and the G/P ratio was 1.32. The clinical neurological symptoms of the pediatric patient during her first hospitalization were mild. After 11 days of treatment, the pediatric patient was cured and discharged from the hospital.

A total of 20 pediatric patients underwent cranial MRI reexaminations at the age of 6 months, and six of them showed a high signal intensity in the bilateral globus pallidus on T_2_WI accompanied by a reduced volume of the globus pallidus. Among them, four showed a high signal intensity of globus pallidus on T_2_WI in the neonatal period. No significant abnormal signals were found in 14 cases, as shown in Table 2 and Figure 2.

**Table 2:** MRI signal manifestations in the KSD and good prognosis groups at 6 months.

Group	*N*	T_1_WI		T_2_WI		Reexamination T_2_WI	
		Hyperintensity	Isointensity	Hyperintensity	Isointensity	Hyperintensity	Isointensity
KSD	12	10	2	4	8	6	6
Good prognosis	8	5	3	0	8	0	8

Follow-up was continued for the 17 patients in the KSD group. A total of 14 pediatric patients were followed up at the age of 1 year, including 11 pediatric patients who underwent cranial MRI reexaminations, and nine pediatric patients were followed up at the age of 2 years, all of whom underwent cranial MRI reexaminations. In the group of 1-year-old pediatric patients, 11 pediatric patients underwent cranial MRI reexamination, and five of them showed symmetrical abnormally long T2 signals in the globus pallidus, among whom three cases showed symmetrical abnormally long T2 signals in the globus pallidus at the first and 6-month-old reexaminations. All nine pediatric patients in the 2-year-old group underwent cranial MRI reexaminations, and three of them showed symmetrical abnormally long T2 signals in the globus pallidus, with development assessments carried out using the Gesell Developmental Schedules and suggesting a KSD. In addition, the three pediatric patients in the 2-year-old group showed symmetrical abnormally long T2 signals in the globus pallidus at the first examination, as well as during the follow-ups at the ages of 6 months and 1 year, as shown in Table 3. These three patients all had severe symptoms of cerebral palsy, hearing loss, and intellectual disability.

**Table 3:** MRI signal manifestations of pediatric patients followed up at the ages of 1 and 2 years.

Group	*N*	T_1_WI	T_1_WI	T_2_WI	T_2_WI
		Hyperintensity	Isointensity	Hyperintensity	Isointensity
1-year-old	11	0	11	5	6
2-year-old	9	0	9	3	6

### Analysis results of G/P ratio and age in days among the groups

**Figure 3:**
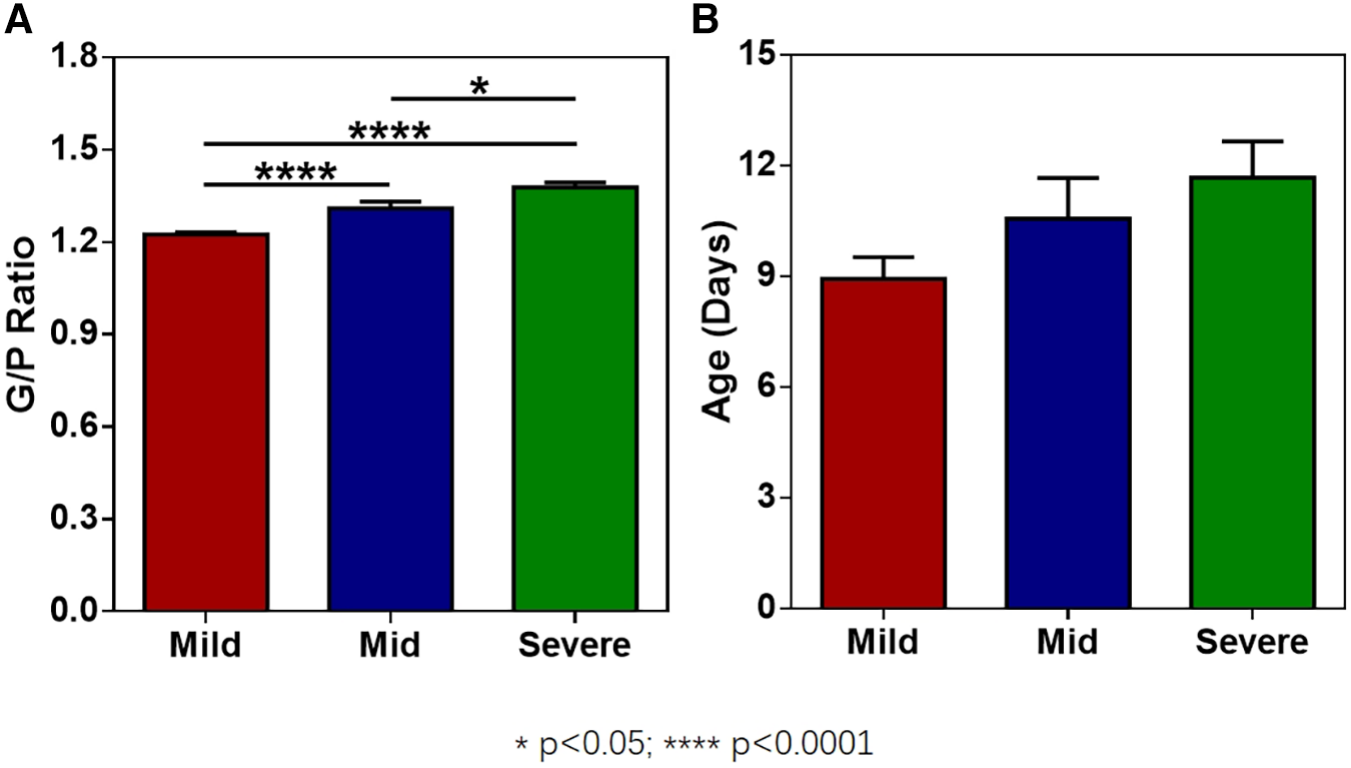
(**A**) The G/P ratios of pediatric patients in the mild, moderate, and severe ABE groups. (**B**) The ages in days of pediatric patients in the mild, moderate, and severe ABE groups at the time of initial MRI examination.

**Figure 4:**
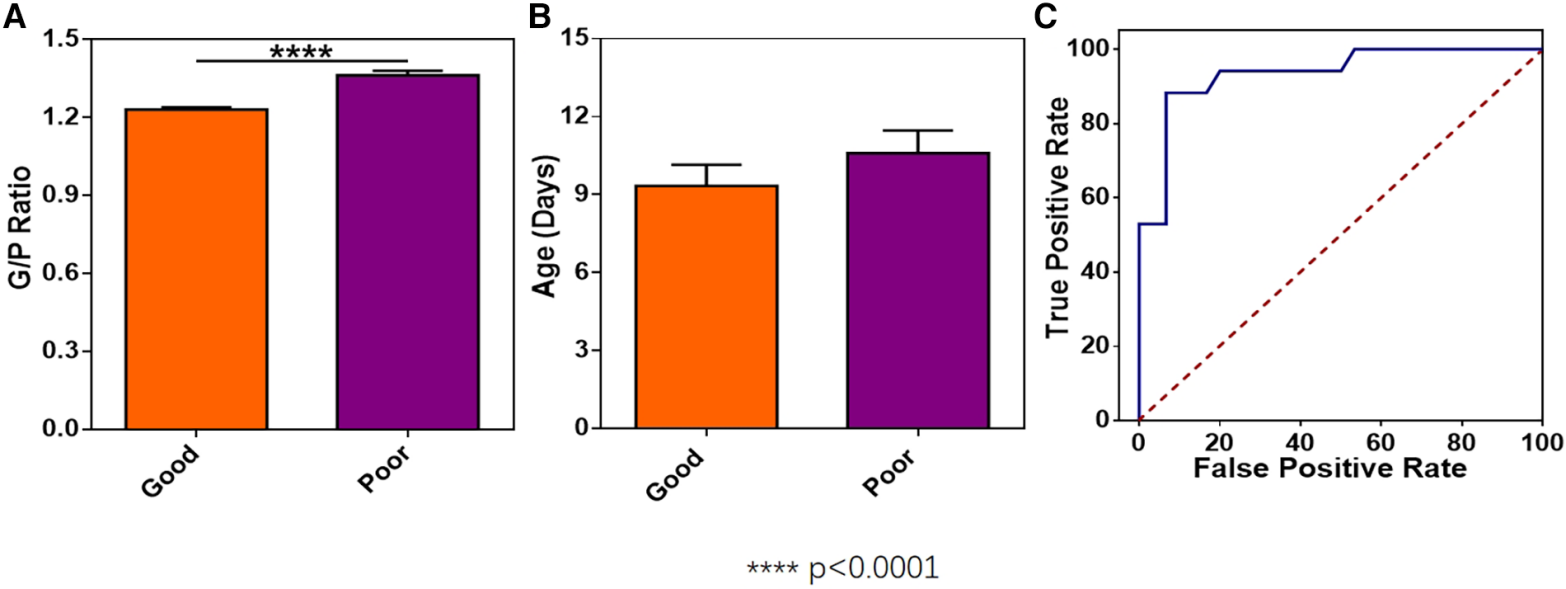
(**A**) The difference in G/P ratio between the KSD group and the good prognosis group. (**B**) The ages in days between the KSD group and good prognosis group at the time of initial MRI examination. (**C**) Receiver operating characteristic curve of the G/P ratio in the KSD group.

The G/P ratios of pediatric patients in the mild, moderate, and severe ABE groups were analyzed, and the differences among the groups were statistically significant (Figure 3A). The G/P ratio in the severe group was the highest, followed by the moderate group and then the mild group. The ages in days of pediatric patients among the three groups were analyzed, and the *P-*value was 0.12, without a statistically significant difference, as shown in Figure 3B and Table 4. The difference in G/P ratio between the KSD group and good prognosis group was statistically significant (Figure 4A). The G/P ratio in the KSD group was significantly higher than that in the good prognosis group. The ages in days of pediatric patients between the two groups were analyzed, and the *P*-value was 0.326, without a statistically significant difference, as shown in Figure 4B and Table 5. When analyzing the G/P ratio in the KSD group, the area under the ROC curve was 93.5%, the optimal critical point was 1.29, the sensitivity was 88.2%, and the specificity was 93.3% (Figure 4C). A KSD was considered when the G/P ratio was greater than 1.29.

**Table 4:** Comparison of G/P ratio and age at the time of initial MRI examination (in days) among the three groups ($\bar{x}\pm s$).

Group	*N*	G/P ratio	Age in days
Mild ABE	50	1.22 ± 0.04	8.92 ± 4.20
Moderate ABE	18	1.31 ± 0.09	10.56 ± 4.69
Severe ABE	9	1.38 ± 0.05	11.7 ± 2.96
*P*-value		0.00	0.12

**Table 5:** Comparison of G/P ratio and age at the time of initial MRI examination (in days) between the two groups ($\bar{x}\pm s$).

Group	N	G/P ratio	Age in days
KSD	17	1.35 ± 0.08	10.59 ± 3.61
Good prognosis	30	1.23 ± 0.05	9.33 ± 4.44
*t* value		7.4	1.0
*P*-value		0.00	0.326

## Discussion

### Value of G/P ratio in the grading of acute bilirubin encephalopathy

Bilirubin can enter cerebrospinal fluid and brain tissue through an incomplete blood–brain barrier, incurring damage to neurons and astrocytes (22, 23). In particular, the globus pallidus is most significantly damaged while the putamen is not affected. Studies have shown that bilateral symmetrical globus pallidus T_1_WI hyperintensity is the characteristic manifestation of neonatal acute bilirubin encephalopathy, which is consistent with the results of this study (24). The results showed that the more severe the neurological symptoms, the higher the intensity of globus pallidus T_1_WI signals, with statistical significance. The research conclusions of Mao et al. suggested that symmetrical hypersignals of globus pallidus on MRI T_1_WI were closely related to the severity and exposure time of hyperbilirubinemia and were an important manifestation of neonatal ABE (25).

Increased globus pallidus signals on T_1_WI are closely related to acute bilirubin encephalopathy. In this study, the putamen was selected for comparisons. Calculating the globus pallidus/putamen signal intensity ratio can eliminate errors in signal intensity value caused by errors in image background and can objectively assess the degree of increase in globus pallidus signals on T_1_WI. The results showed that the globus pallidus/putamen T_1_WI signal intensity ratio (G/P ratio) was closely related to clinical grading. The more severe the clinical grading, the higher the G/P ratio (as shown in Table 3), suggesting a more severe damage to the nuclei of the globus pallidus, thereby providing an imaging basis for clinical grading.

The toxic effect of bilirubin on the nervous system is reversible at an early stage, so T_1_WI hyperintensity in this acute phase may be a transient phenomenon, and the prognosis of disease is relatively good. In this study, the 47 pediatric patients who underwent a follow-up reexamination at the age of 6 months were divided into the good prognosis group and the KSD group according to the results of follow-up reexaminations. The G/P ratio in their acute phase was assessed. The results showed that there was a statistically significant difference in the G/P ratio between the good prognosis group and the KSD group. The ROC curve of the KSD group was predicted by analyzing the G/P ratio. The area under the curve was 0.935, and the optimal critical point was 1.29. Therefore, when the G/P ratio was greater than 1.29, the damage to the globus pallidus was serious, and this could be used as an important basis for the clinical prognosis assessment of bilirubin encephalopathy (Figure 4).

In this study, six of the 20 pediatric patients who were reexamined at the age of 6 months showed high signals in the globus pallidus on T_2_WI, and the volume of the globus pallidus was reduced. At the same time, all also developed KSD. Among them, five showed dyskinetic cerebral palsy, which is consistent with the finding of previous research whereby high signals in the globus pallidus on T_2_WI suggest a KSD (15). This may be related to bilirubin causing necrosis and the demyelination of many neurons, accompanied by glial cell hyperplasia, eventually remaining as irreversible damage. This is consistent with the persistently high signals in the globus pallidus on T_2_WI at the subsequent follow-ups at the ages of 1 and 2 years. In this study, four pediatric patients showed high signals in the globus pallidus on T_1_WI and T_2_WI in their initial MRI scans. In the follow-up reexamination of these cases, it was found that, except for one case who was lost to follow-up at the age of 2 years, there were residual high signals in the globus pallidus on T_2_WI in every cranial MRI examination, and there were serious clinical sequelae, with all cases having serious symptoms of cerebral palsy, hearing loss, and intellectual disability.

## Conclusions

In summary, this work investigated the relationship between the G/P ratio in MR images and the ABE in neonates and confirmed that the G/P ratio could assist in the clinical grading of ABE. Moreover, the G/P ratio was also proved capable to indicate the damage degree of the globus pallidus and assist the assessment of a prognosis. Specifically, a G/P ratio greater than 1.29 indicated that the globus pallidus was seriously damaged and the prognosis of pediatric patients was often poor. The G/P ratio, which can be obtained by simple measurement and calculation in conventional magnetic resonance sequences, is a simple operation with strong reproducibility and has great clinical practical value in the grading and prognosis assessment of neonatal ABE.

## Data Availability

The original contributions presented in the study are included in the article/Supplementary Material, further inquiries can be directed to the corresponding author.

## References

[1] Hamza A. Kernicterus. Autops Case Rep. (2019) 9(1):e2018057. 10.4322/acr.2018.057 30863731 PMC6394357

[2] Riordan SM Shapiro SM. Review of bilirubin neurotoxicity I: molecular biology and neuropathology of disease. Pediatr Res. (2020) 87(2):327–31. 10.1038/s41390-019-0608-0 31600770

[3] Olusanya BO Kaplan M Hansen WR. Neonatal hyperbilirubinemia: a global perspective. Lancet Child Adolesc Health. (2018) 2:610–20. 10.1016/S2352-4642(18)30139-1 30119720

[4] Conti CP S. Bilirubin: the toxic mechanisms of an antioxidant molecule. Arch Argent Pediatr. (2021) 119(1):e18–25. 10.5546/aap.2021.eng.e18 33458986

[5] Dong XY Wei QF Li ZK Gu J Meng DH Guo JZ Causes of severe neonatal hyperbilirubinemia: a multicenter study of three regions in China. World J Pediatr. (2021) 17(3):290–7. 10.1007/s12519-021-00422-3 34047994

[6] Newman TB Liljestrand P Jeremy RJ Ferriero DM Wu YW Hudes ES Outcomes among neonates with total serum bilirubin levels of 25 mg per deciliter or more. N Engl J Med. (2006) 354:1889–900. 10.1056/NEJMoa054244 16672700

[7] Usman F DU Shapiro SM Le Pichon JB Slusher TM. Acute bilirubin encephalopathy and its progression to kernicterus: current perspectives. Res Rep Neonatol. (2018) 8:33–44.

[8] Falcao AS Silva RF Pancadas S Fernandes A Brito MA Brites D. Apoptosis and impairment of neurite network by short exposure of immature rat cortical neurons to unconjugated bilirubin increase with cell differentiation and are additionally enhanced by an inflammatory stimulus. J Neurosci Res. (2007) 85(6):1229–39. 10.1002/jnr.21227 17342778

[9] Iskander I Gamaleldin R El Houchi S El Shenawy A Seoud I El Gharbawi N Serum bilirubin and bilirubin/albumin ratio as predictors of bilirubin encephalopathy. Pediatrics. (2014) 134:e1330–9. 10.1542/peds.2013-1764 25332491 PMC4210789

[10] Luo F Chen Z Lin H Wang C Ma X Shi L. Evaluation of cerebral function in high risk term infants by using a scoring system based on aEEG. Transl Pediatr. (2014) 3:278–86. 10.3978/j.issn.2224-4336.2014.10.03 26835347 PMC4728834

[11] Olds C Oghalai JS. Audiologic impairment associated with bilirubin-induced neurologic damage. Semin Fetal Neonatal Med. (2015) 20(1):42–6. 10.1016/j.siny.2014.12.006 25575899 PMC4314954

[12] Wu H Li Z Liu J Liu G Yang X. Clinical study on amplitude integrated electroencephalogram in cerebral injury caused by severe neonatal hyperbilirubinemia. Minerva Pediatr. (2018) 70(6):539–44. 10.23736/S0026-4946.17.04792-2 28206723

[13] Cinar A Yildirim M. Detection of tumors on brain MRI images using the hybrid convolutional neural network architecture. Med Hypotheses. (2020) 139. 10.1016/j.mehy.2020.109684 32240877

[14] Jia G Gong J Ding H Li A Wang J Xu J. Logistic regression analysis on risk factors of neonates T1WI hyperintensity at globus pallidus and subthalamic nucleus. Zhonghua Yi Xue Za Zhi. (2015) 95:1171–4. 26081363

[15] Sugama S Soeda A Eto Y. Magnetic resonance imaging in three pediatric patients with kernicterus. Pediatr Neurol. (2001) 25:328–31. 10.1016/s0887-8994(01)00306-x. 11704404

[16] Sari S Yavuz A Batur A Bora A Caksen H. Brain magnetic resonance imaging and magnetic resonance spectroscopy findings of pediatric patients with kernicterus. Pol J Radiol. (2015) 80:72–80. 10.12659/PJR.892643 25745520 PMC4327183

[17] Wu W Zhang P Wang X Chineah A Lou M. Usefulness of (1) H-MRS in differentiating bilirubin encephalopathy from severe hyperbilirubinemia in neonates. J Magn Reson Imaging. (2013) 38:634–40. 10.1002/jmri.23995 23440930

[18] Shapiro SM. Definition of the clinical spectrum of kernicterus and bilirubin-induced neurological dysfunction (BIND). J Perinatol. (2005) 25:54–9. 10.1038/sj.jp.7211157 15578034

[19] Bhutani VK Johnson-Hamerman L. The clinical syndrome of bilirubin-induced neurologic dysfunction. Semin Fetal Neonatal Med. (2015) 20(1):6–13. 10.1016/j.siny.2014.12.008 25577653

[20] Ball RS. The Gesell developmental schedules: Arnold Gesell (1880-1961). J Abnorm Child Psychol. (1977) 5:233–9. 10.1007/bf00913694 332745

[21] Cai YJ Song YY Huang ZJ Li J Qi JY Xiao XW Effects of postnatal growth retardation on early neurodevelopment in premature infants with intrauterine growth retardation. Zhongguo Dang Dai Er Ke Za Zhi. (2015) 17:893–7. 26412165

[22] Silva RF Rodrigues CM Brites D. Rat cultured neuronal and glial cells respond differently to toxicity of unconjugated bilirubin. Pediatr Res. (2002) 51:535–41. 10.1203/00006450-200204000-00022 11919342

[23] Hansen TWR Wong RJ Stevenson DK. Molecular physiology and pathophysiology of bilirubin handling by the blood, liver, intestine, and brain in the newborn. Physiol Rev. (2020) 100(3):1291–346. 10.1152/physrev.00004.2019 32401177

[24] Hori S Taoka T Ochi T Miyasaka T Sakamoto M Takayama K Structures showing negative correlations of signal intensity with postnatal age on T1-weighted imaging of the brain of newborns and infants. Magn Reson Med Sci. (2017) 16(4):325–31. 10.2463/mrms.mp.2015-0168 28202853 PMC5743524

[25] Mao J Chen LY Fu JH Li J Xue XD. Clinical evaluation by MRI on the neonate infants with hypoglycemic brain damage. Zhonghua Er Ke Za Zhi. (2007) 45:518–22. 17953809

